# What Makes Musical Prodigies?

**DOI:** 10.3389/fpsyg.2020.566373

**Published:** 2020-12-11

**Authors:** Chanel Marion-St-Onge, Michael W. Weiss, Megha Sharda, Isabelle Peretz

**Affiliations:** Department of Psychology, International Laboratory for Brain, Music, and Sound Research, University of Montreal, Montreal, QC, Canada

**Keywords:** musical prodigies, musical talent, expertise, achievement, practice, intelligence, personality

## Abstract

Musical prodigies reach exceptionally high levels of achievement before adolescence. Despite longstanding interest and fascination in musical prodigies, little is known about their psychological profile. Here we assess to what extent practice, intelligence, and personality make musical prodigies a distinct category of musician. Nineteen former or current musical prodigies (aged 12–34) were compared to 35 musicians (aged 14–37) with either an early (mean age 6) or late (mean age 10) start but similar amount of musical training, and 16 non-musicians (aged 14–34). All completed a Wechsler IQ test, the Big Five Inventory, the Autism Spectrum Quotient, the Barcelona Music Reward Questionnaire, the Dispositional Flow Scale, and a detailed history of their lifetime music practice. None of the psychological traits distinguished musical prodigies from control musicians or non-musicians except their propensity to report flow during practice. The other aspects that differentiated musical prodigies from their peers were the intensity of their practice before adolescence, and the source of their motivation when they began to play. Thus practice, by itself, does not make a prodigy. The results are compatible with multifactorial models of expertise, with prodigies lying at the high end of the continuum. In summary, prodigies are expected to present brain predispositions facilitating their success in learning an instrument, which could be amplified by their early and intense practice happening at a moment when brain plasticity is heightened.

## Introduction

CH plays the violin exceptionally well. He’s a 26-year-old acclaimed professional musician who studied at Juilliard, has won numerous national and international competitions, and currently plays on a Stradivarius violin. He made his orchestral debut at 7 years old. A musician like CH, who showed “superior performance within a specific domain” before adolescence, is considered to be a musical prodigy in the present study (see Supplementary Table 1 for definitions). Here, in the largest sample of exceptional musicians considered so far, we examine non-musical traits, such as practice, autistic traits, and intelligence, that have been associated with musical prodigiousness.

**FIGURE 1:**
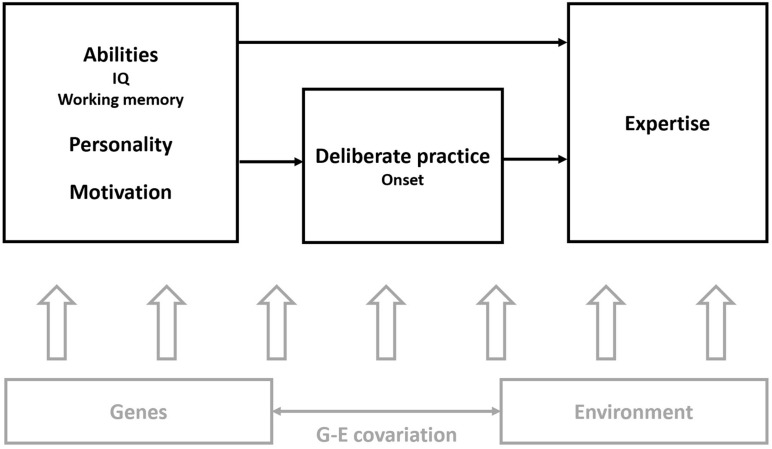
Adaptation from the Multifactorial Gene–Environment Interaction Model proposed by Ullén et al. (2016) in which the factors assessed here are highlighted in black. The arrows represent the influence between psychological traits, practice behavior, and expertise (or achievement). Below are the complex influences of genes, environment, and their interaction on all the variables depicted above.

In doing so, we endorse the Multifactorial Gene–Environment Interaction Model proposed by Ullén et al. (2016) (Figure 1), which assumes complex interactions between genes, environment, practice behavior, and psychological traits (Mosing et al., 2014).

Practice is obviously central to the development of any skill, and musical skill in particular. From the influential deliberate practice perspective, practice is the only important factor in acquiring expertise (Ericsson et al., 1993). Other perspectives hold that practice alone is not sufficient. In a meta-analysis on the relationship between practice and performance, Macnamara et al. (2014) found that the variance in music performance explained by deliberate practice is 21%, which leaves the majority of variance unexplained. Complicating matters further, individuals vary considerably in the amount of practice needed to reach expert-level performance (Ackerman, 2014). For example, in chess, the minimum amount of deliberate practice required to achieve master level is around 3,000 h, but some players accumulate as many as 20,000 h without reaching that status (Campitelli and Gobet, 2011). Thus, the relation between practice and performance is not straightforward.

Practice is not a purely environmental factor. Genetic predispositions also come into play. There is no difference, for example, in music perception abilities of monozygotic twins with differing amounts of musical practice (Mosing et al., 2014). The age of onset of musical training can also interact with genetic differences in brain structure and function (Herholz and Zatorre, 2012). A confluence of neurogenetic factors might influence practice, as well as musical abilities like the precision of motor timing in sequential tapping, complicating the relationship between practice and musical achievement (Ullén et al., 2015).

The Multifactorial Gene–Environment Interaction Model (MGIM; Ullén et al., 2016; Figure 1 for an adapted version) of music proficiency and expertise is arguably the most comprehensive model of musical talent proposed so far. The model is evidence-driven in the sense that it emerges from recent findings in the field of expertise. It incorporates the roles of multiple factors in expertise development, such as practice required to reach a certain level of performance, personality traits, IQ, and working memory.

Motivation to practice is another psychological dimension considered in the model suggested by Ullén et al. (2016), but often ignored in neurogenetic studies of musicality. This trait seems especially relevant to prodigies, who have been described as possessing a “rage to master,” or a drive fueling their interest and capacity to practice for extended periods of time (Winner, 2000). As Gagné and McPherson (2016) note, the terminology used by Winner encompasses various concepts such as flow, obsessive passion, and intrinsic motivation. Indeed, the tendency to experience flow may contribute to prodigies’ motivation. Flow is a psychological state characterized by intense concentration and a heightened sense of control, and it constitutes an experience that is inherently rewarding (Nakamura and Csikszentmihalyi, 2009). The experience of flow when playing music correlates with amount of music practice (Butkovic et al., 2015; Marin and Bhattacharya, 2013), but flow itself is not a predictor of achievement (i.e., which musician will win a competition; Marin and Bhattacharya, 2013). Moreover, personality traits like openness to experience and musical flow share genetic influence (Butkovic et al., 2015). Accordingly, intrinsic motivation, frequency of practice, propensity to experience flow during practice, and reward experienced with music, will be examined here.

Besides practice and motivation, the presence of autistic traits could distinguish prodigies from their peers. Autistic traits are measured by metrics such as the Autism Spectrum Quotient (AQ; Baron-Cohen et al., 2001). A defining autistic trait is attention to detail, which refers to the propensity to focus attention on detailed aspects of sensory information, and which may be more prevalent among musical prodigies (Ruthsatz and Urbach, 2012). Because autistic traits are independent from the personality components of the Big Five inventory (Wakabayashi et al., 2006; Austin, 2005), all participants in the present study will complete the Autism Spectrum Quotient questionnaire in addition to the Big Five Inventory.

Enhanced intelligence is another trait often associated with musical training (for reviews, Schellenberg and Weiss, 2013; Miendlarzewska and Trost, 2014; Swaminathan and Schellenberg, 2019), but most research has focused on typical musicians. Whether musical prodigies, who represent the extreme of musical achievement, would obtain correspondingly high IQ scores is unclear. Support for this idea comes from the study of a relatively large sample of prodigies (*n* = 18), of which eight were musical prodigies. The musical prodigies obtained a high IQ (*M* = 129) compared to the general population, with especially high scores for working memory (Ruthsatz et al., 2014). A more recent case study conducted with a musical prodigy also showed superior working memory (Comeau et al., 2018).

Empirical research on musical prodigies is scarce. Case studies have investigated aspects of musical and cognitive abilities in individual musical prodigies (Comeau et al., 2018; Ruthsatz and Detterman, 2003; Dalla Bella et al., 2016). The typical method compares a prodigy to a control group matched on age or musical training, or uses normalized tests rather than a control group. For example, there are many reports of prodigies who possess absolute pitch – the ability to automatically identify a note without prior reference (Gagné and McPherson, 2016). However, its prevalence in prodigies relative to non-prodigy musicians has not been empirically assessed (Comeau et al., 2018). To our knowledge only one research group has recruited multiple prodigies for study, and these samples were recruited across different domains of expertise (e.g., music, visual arts, and maths), and were not compared to a control group (Ruthsatz and Urbach, 2012; Ruthsatz et al., 2014). No study to date has compared a group of musical prodigies to control groups matched on musical experience.

In the present study, we assess the extent to which prodigious talent exists on a continuum with the trajectory of typical musicians, or alternatively, constitutes a distinct category. We may assume that predispositions play an outsized role in the achievements of prodigies because they achieve so much so early in life, but the nature of those predispositions and their link with behavior and eventual achievement is unknown. In keeping with the MGIM framework (Ullén et al., 2016), we ask whether the prodigies’ expertise (or achievement) is influenced by psychological traits like cognitive abilities, personality, motivation, and deliberate practice behavior, and whether there is a link between practice and psychological traits. We also consider, as an alternative view, whether the prodigy phenomenon can be explained by a simpler framework such as deliberate practice (Ericsson et al., 1993).

The study of prodigies may help to identify which ingredients are critical to reach exceptional performance in typical musicians. To answer these questions, we compared four groups of adolescent or adult participants, former or current prodigies, musicians who started training early in childhood, musicians who started training later in childhood, and non-musicians.

## Materials and Methods

### Participants

We recruited 19 current or former prodigies. Six of them were aged 12 to 14 at the moment of testing and 13 were adult participants who were prodigies in their youth (hereafter, *prodigies*). They were recruited through online searches, references from professional musicians and music teachers, and public announcements. Detailed demographic and musical experience information are listed in Tables 1, 2, respectively. Classification as prodigy was established by meeting at least one of the following criteria before age 14: (1) high achievement in performance, like winning a first prize in a national or international competition, or winning multiple regional competitions, or (2) special recognition of talent through television or documentary appearances, or orchestral debut (as used in Ruthsatz and Urbach, 2012). Their achievements, listed in Supplementary Table 2, were confirmed in a semi-structured interview. Two prodigies (siblings) were diagnosed with autism spectrum disorder early in life.

**TABLE 1:** Demographics.

Group	Prodigies	Early-trained	Late-trained	Non-musicians	Statistics
*N*	19	16	19	16	
Sex (*F* = female; *M* = male)	7 F, 12 M	7 F, 9 M	7 F, 12 M	9 F, 7 M	*X*^2^(3, *N* = 70) = 1.74, *p* = 0.628
Age (years)	21.3 ± 7.4 (12–34)	23.3 ± 6.2 (14–33)	25.2 ± 7.0 (14–37)	24.4 ± 6.9 (14–36)	*F*(3,66) = 1.10, *p* = 0.356
Education (years)	14.0 ± 5.0 (6–21)	15.4 ± 4.0 (8–21)	16.8 ± 4.3 (8–25)	16.9 ± 3.9 (9–25)	*F*(3,66) = 1.82, *p* = 0.153

*Values are reported in mean ± standard deviation with range in parentheses.*

**TABLE 2:** Musical experience.

Group	Prodigies	Early-trained	Late-trained	Statistics
*N*	19	16	19	
Age of onset (years)	4.9 ± 1.3 (3–8)	5.5 ± 1.5 (4–9)	10.3 ± 2.5 (7–15)	*F*(2,51) = 46.59, *p* < 0.001
Musical experience (years)	17.2 ± 7.6 (8–31)	18.1 ± 6.4 (9–28)	15.2 ± 6.5 (7–28)	*F*(2,51) = 0.84, *p* = 0.438
Lifetime practice (hours)	12,710 (836–35,788)	11,576 (628–34,192)	11,005 (732–50,372)	*F*(2,51) = 0.13, *p* = 0.876

*Values are reported in mean ± standard deviation with range in parentheses.*

There were three control groups, with each group differing in their musical experience. Early-trained musicians (*N* = 16; hereafter, *early-trained*) were similar to prodigies in age of onset of musical training and years of musical experience but did not show exceptional talent before the age of 14. Late-trained musicians (*N* = 19; hereafter, *late-trained*) began to play their instrument later than the prodigies and early-trained musicians, on average, while accumulating a similar number of years of musical training at the time of testing. Early-trained musicians were matched individually to prodigies on age of onset of musical experience (±2 years). Late-trained musicians had a delayed onset of training after age 7 and were also matched on years of musical experience. Before 18 years old, the majority of control musicians (30 out of 35 control musicians) did not report any achievements such as those considered for the prodigy criteria.

During the interview conducted with each musician, we collected practice data on the daily or weekly estimated number of hours of deliberate practice. For participants under age 16, parents were present during the interview. Yearly estimated number of hours of practice were calculated by summing the number of hours of daily or weekly practice reported by each participant for each year of musical experience, as in other research (Ericsson et al., 1993). For example, if a participant reported practicing 20 min per day and 6 days per week, this amounts to 2 h per week for 52 weeks, and 104 h for that particular year. For each musician, we also calculated accumulated deliberate practice by summing the yearly amount of practice from the onset of musical experience. Detailed information is listed in Table 2.

Sixteen non-musicians who had less than three years of musical experience and were not currently active musically were also tested. All non-musicians performed within the normal range on the online test for the evaluation of amusia (Peretz and Vuvan, 2017). Because musical aptitude may vary among non-musicians, we used a test of basic musical perception skills, the Musical Ear Test (Wallentin et al., 2010). Non-musicians obtained a mean of 72.2% correct (SD = 11.9) in the melody perception subtest and a mean of 72.7% correct (SD = 8.3) in the rhythm perception subtest. Their performance is comparable to the non-musicians in the original paper, with means of 69.7% (SD = 11.1) and 70.6% (SD = 8.0), respectively (Wallentin et al., 2010).

Other factors known to affect performance on behavioral tests and questionnaires, such as age, sex, and education, were matched across all groups (see Table 1). Most of the sample was Caucasian (48 out of 70). Seven out of 19 prodigies reported being of Asian ethnicity (South or East).

Due to time constraints and early changes in the protocol, there is missing data for one late-trained musician (Barcelona Music Reward Questionnaire), one early-trained musician (visual working memory), and one prodigy (motivation). Moreover, one prodigy and one late-trained musician were administered an abbreviated version of the IQ measure (WASI) instead of the full-scale IQ (WAIS-IV) because of time constraints. Accordingly, IQ index values are unavailable for these two participants. There are missing data for 8 participants on the measure of flow (2 prodigies, 4 early-trained, and 2 late-trained), because the measure was administered remotely and some did not reply.

### Materials and Procedure

#### Online Questionnaire

Prior to their lab visit, participants completed an online questionnaire. The first section contained consent and demographics information. The online questionnaire also contained sections on absolute pitch, reward, motivation to play their instrument, and personality traits (see descriptions below). For participants who were minors, parents completed the consent form and demographics information; the remaining sections were completed by the participants themselves.

#### Reward, Motivation, and Flow Questionnaires

The Barcelona Music Reward Questionnaire (BMRQ; Mas-Herrero et al., 2013) consists of 22 questions that assess reward associated to music in five dimensions: music seeking (e.g., *I’m always looking for new music*), emotion evocation (e.g., *I get emotional, listening to certain pieces of music*), mood regulation (e.g., *Music helps me chill out*), social reward (e.g., *Music makes me bond with other people*), and sensory-motor (e.g., *Music often makes me dance*). Answers were provided on a 5-point Likert scale, with 1 meaning *Completely disagree* and 5 meaning *Completely agree*.

To assess musicians’ motivation to play their instrument, we selected items from the questionnaire of Desrochers et al. (2006) which did not exhibit floor or ceiling effects (i.e., with a rate equal or lower than 40% of extreme values). These items are listed in Table 3.

**TABLE 3:** Selected items to measure motivation.

Item	Response scale		
I play my instrument…			
Because I would feel guilty if I did not do it	Totally disagree	1 2 3 4 5	Totally agree
Because it adds something special to my personality	Totally disagree	1 2 3 4 5	Totally agree
What was the source of motivation when you began to play your instrument?	Completely internal	1 2 3 4 5	Completely external

In addition, most participants filled a questionnaire assessing flow during musical practice, the Dispositional Flow Scale 2 (Jackson and Eklund, 2004). This questionnaire consists of 36 items assessing flow, using a 5-point scale (1 = *never* to 5 = *always*). The global score was obtained by calculating the mean score of all items. Examples of items, all following the statement “*When I practice my instrument*…,” include: “*My attention is focused entirely on what I am doing*,” “*I really enjoy the experience*,” “*It feels like time goes by quickly*,” “*I am challenged, but I believe my skills will allow me to meet the challenge*.”

#### Personality Traits

The Autism Spectrum Quotient (AQ; Baron-Cohen et al., 2001) consists of 50 items meant to measure five dimensions of the autistic profile: social skill (e.g., *I would rather go to a library than a party*), attention switching (e.g., *I prefer to do things the same way over and over again*), attention to detail (e.g., *I tend to notice details that others do not*; *I am fascinated by numbers*), communication (e.g., *I frequently find that I don’t know how to keep a conversation going*), and imagination (e.g., *I find it difficult to imagine what it would be like to be someone else*). Answers were provided using a scale with four options: *definitely agree*, *slightly agree*, *slightly disagree* and *definitely disagree*.

The Big Five Inventory (John et al., 1991) contains 45 questions constructed to measure five different dimensions of personality: openness to experience (e.g., *Likes artistic and creative experiences*), conscientiousness (e.g., *Does things carefully and completely*), extraversion (e.g., *Is outgoing, sociable*), agreeableness (e.g., *Is considerate and kind to almost everyone*), and neuroticism (e.g., *Worries a lot*). Answers are provided using a 5-point Likert scale, with 1 meaning *Disagree strongly* and 5 meaning *Agree strongly*.

#### Intellectual Quotient and Working Memory

Standardized tests of intellectual quotient (IQ) were administered to all participants. For musicians, the Wechsler Adult Intelligence Scale – Fourth Edition (WAIS-IV; Wechsler, 2008) was administered to participants aged 17 or older, and the Wechsler Intelligence Scale for Children – Fourth Edition (WISC-IV; Wechsler, 2003) was administered to participants aged 16 or younger. These batteries provide a global IQ score as well as 4 index scores: verbal comprehension, perceptual reasoning, working memory, and processing speed. For non-musicians, an abbreviated measure of IQ was used, the Wechsler Abbreviated Scale of Intelligence (WASI; Wechsler, 1999) with 2 (*N* = 10) or 4 subtests (*N* = 15), which provide a full-2 or full-4 IQ score, respectively. WASI versions vary because the protocol was changed for time-saving purposes. The WASI was used for non-musicians because IQ is well known in the normal population, obviating the need for more extensive evaluation. Global IQ and indices for the WAIS-IV and WISC-IV were calculated using the summation of the subtests administered, and normed using the age-appropriate tables of the WAIS-IV, WISC-IV, and WASI. The mean in the normal population is 100 points, and one standard deviation corresponds to 15 points.

Since the WAIS-IV subtests of working memory are only auditory-verbal and because visual working memory could be involved in music learning (e.g., in sight-reading; Meinz and Hambrick, 2010), all participants completed a test of spatial working memory from the Wechsler Memory Scale, Third Edition (WMS-III; Wechsler, 2001). In this task, the experimenter points to blocks on a plank in a specific sequence, and the participant must point to them in the same order. The procedure is repeated with the instruction to point in the reverse order. The number of blocks increases until the participant errs. Raw scores (i.e., number of sequences correctly recalled) were calculated and used in the analyses, with higher scores indicating better spatial working memory.

The tests were administrated individually in a quiet, closed room on the campus of the University of Montreal.

## Results

**FIGURE 2:**
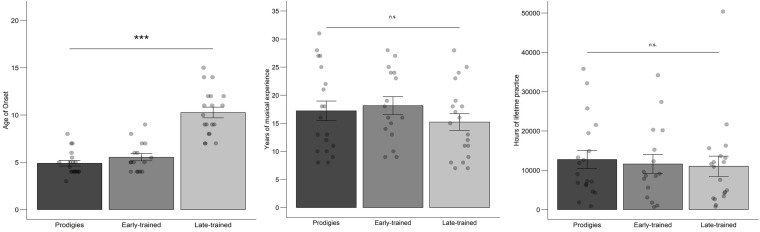
Musical experience measures: mean, standard error and individual data by group. Points are jittered horizontally for visualization purposes.

**FIGURE 3:**
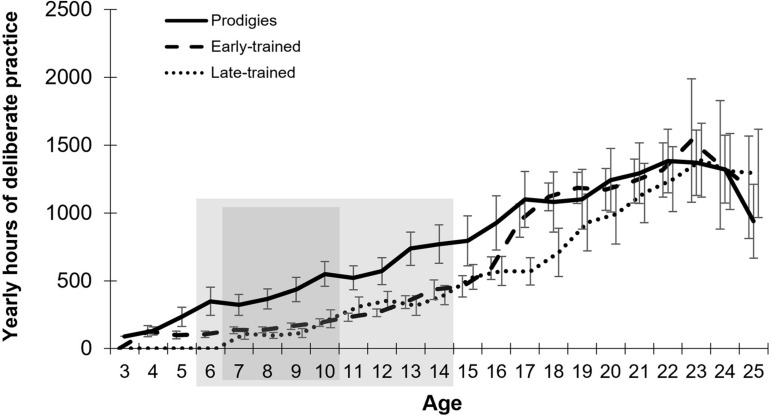
Mean yearly amount and standard error of deliberate practice as a function of age, by group. Gray areas represent the longest stretches where permutation analysis showed a significant difference between prodigies and early-trained musicians (larger rectangle) and between prodigies and late-trained (smaller rectangle).

Prodigy status was reached at a mean age of 10.3 years (*SD* = 1.8; *range* = 7–13), after a mean of 5.4 years of musical experience (*SD* = 1.3; *range* = 3–8) and an accumulated average amount of practice of 2,364 h, although variability was large (*range* = 187–7,357 h). Individual data are presented in Supplementary Figure 1. At the time of testing, prodigies accumulated a total amount of practice that did not differ statistically from their musician peers (Figure 2). They also reported more frequent practice in childhood than typical musicians (Figure 3). By the cut-off age of 14 for the status of prodigy, prodigies accumulated twice as much practice (*M* = 4,563, *range* = 702–13,252 h) as early-trained musicians (*M* = 2,027, *range* = 378–4,004 h).

**FIGURE 4:**
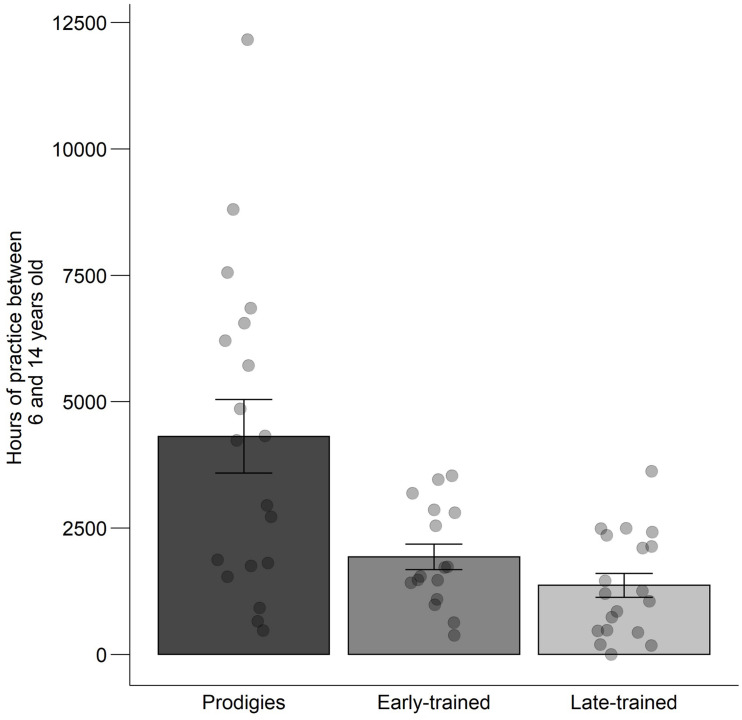
Deliberate practice accumulated between 6 and 14 years old: mean, standard error and individual data, by group. Points are jittered horizontally for visualization purposes.

Group differences in early practice were assessed using permutation analyses. Group attribution was shuffled across participants, and *t*-tests were calculated at each age. The maximum number of consecutive years that obtained a significant group difference (*p* < 0.05) was logged, and the process was repeated 1000 times to obtain a null distribution. The observed results (i.e., 9 years of consecutive, significant differences between prodigies and early-trained musicians; 6–14 years old) were less likely than 99.8% of results in the null distribution. A similar permutation test was conducted by comparing prodigies and late-trained musicians across ages with sufficient data (7–18 years of age). The observed result (i.e., group differences from age 7–10 inclusive or four consecutive years), was less likely than 96% of the null distribution (see gray boxes in Figure 3). These results provide further support that prodigies differed in their practice habits in childhood and early adolescence. Visualization of practice between 6 and 14 years old by individual (Figure 4) shows a large variability in the prodigies group, with around half of participants practicing as much as their age-matched peers, and half practicing more.

**FIGURE 5:**
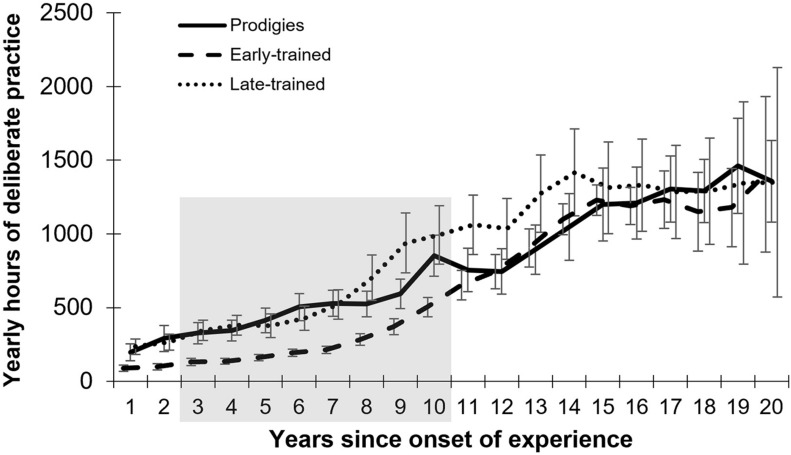
Mean yearly amount of practice and standard error as a function of year since onset of musical experience, by group. The gray area represents the longest stretch where permutation analysis showed a significant difference between prodigies and early-trained musicians.

Since musicians started practicing at different ages, we also analyzed the data by year of musical experience (i.e., years since onset of experience; Figure 5). Using the permutation method outlined above, prodigies were found to accumulate more hours of practice than early-trained musicians from years 3–10 inclusive, thus for eight consecutive years, which corresponds to better performance than 99.5% of the null distribution. In contrast, prodigies did not practice more than late-trained musicians during any year when measured from onset of training.

Almost half of the musicians (*n* = 23 of 54) reported having absolute pitch, with roughly half of that group (*n* = 11) being prodigies. However, the proportion did not differ significantly across groups, with 58% of prodigies (*n* = 11 of 19), 44% of early-trained (*n* = 7 of 16), and 26% of late-trained musicians (*n* = 5 of 19), *X*^2^(2, *N* = 54) = 3.89, *p* = 0.143.

### Musical Reward and Motivation

**FIGURE 6:**
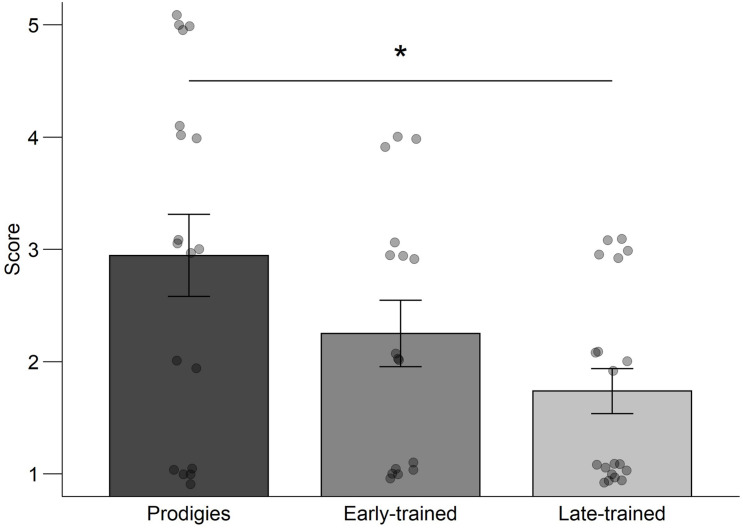
Source of motivation when beginning to play: mean rating score, standard error and individual data, by group. Points are jittered for visualization purposes. See also row three of Table 3.

Prodigies did not report finding music more rewarding than musicians or non-musicians. This was tested with an ANOVA computed on the BMRQ global score with group (prodigies, early-trained, late-trained, non-musicians) as a between-subjects factor, *F*(3,65) = 1.14, *p* = 0.339, η^2^ = 0.050. ANOVAs were computed on the scores from each of the three motivation questions (Table 3), with group (prodigies, early-trained, late-trained) as a between-subjects factor. Responses to the motivation questions “*I play my instrument*… *Because I would feel guilty if I did not do it*” yielded no significant group effect, *F*(2,50) = 1.36, *p* = 0.267, η^2^ = 0.051, and neither did responses to the question “*I play my instrument*… *Because it adds something special to my personality*”, *F*(2,50) = 0.40, *p* = 673, η^2^ = 0.016. However, responses to the question on the source of motivation when beginning to play their instrument showed a significant group effect [*F*(2,50) = 4.48, *p* = 0.016, η^2^ = 0.152; Figure 6]. *Post hoc* pairwise comparisons using Welch’s *t*-test (Bonferroni-Holm correction, three pairwise comparisons between groups) showed that prodigies (*M* = 2.94) reported a more external source of motivation when they started to play their instruments compared to late-trained musicians (*M* = 1.74), *t*(26.45) = 2.90, *p* = 0.022.

**FIGURE 7:**
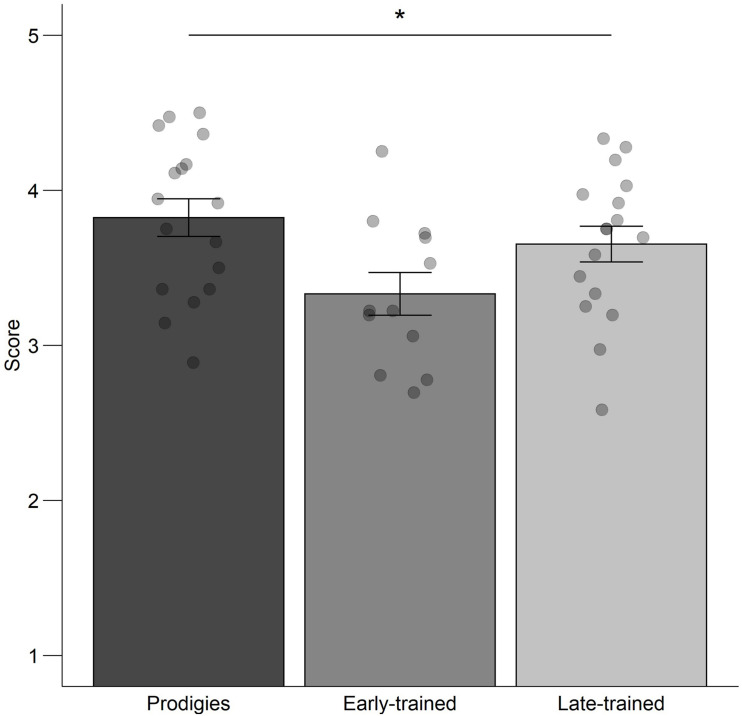
Global flow: mean score, standard error and individual data, by group. Points are jittered horizontally for visualization purposes.

Global flow during music practice varied across groups (Figure 7), as shown by an ANOVA computed on the global flow score with group (prodigies, early-trained, late-trained) as a between-subjects factor, *F*(2,43) = 3.62, *p* = 0.035. *Post hoc* group comparisons showed that prodigies reported significantly more flow when they practice their instrument (*M* = 3.8, *SD* = 0.5) compared to early-trained musicians (*M* = 3.3, *SD* = 0.5, *p* = 0.039, Bonferroni-Holm correction used for three pairwise comparisons between groups). Early-trained musicians did not differ significantly from late-trained musicians (*M* = 3.7, *SD* = 0.5, *p* = 0.173).

### Personality Traits

**FIGURE 8:**
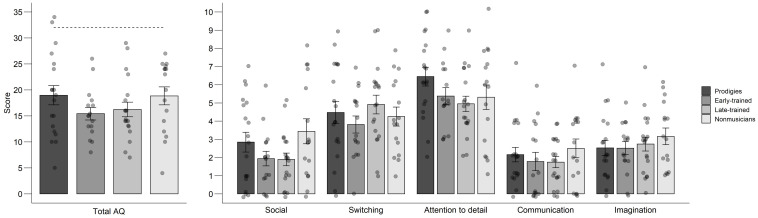
Autism Spectrum Quotient (AQ) scores. In the left panel, mean scores, standard error and individual data for the total AQ score, by group. The dashed line indicates the cut-off score for clinically significant levels of autistic traits; In the right panel, mean scores, standard error and individual data by dimension and group. Points are jittered for visualization purposes.

There was no indication that prodigies, as a group, possessed more autistic traits than other musicians (Figure 8). The ANOVA computed on the AQ scores with group (prodigies, early-trained, late-trained, non-musicians) as a between-subjects factor and dimension (social, attention switching, attention to detail, communication, and imagination) as a within-subject factor did not reveal an effect of group, *F*(3,66) = 1.28, *p* = 0.289, $\eta_{p}^{2}$ = 0.04. A dimension effect was significant, *F*(4,264) = 51.18, *p* < 0.001, $\eta_{p}^{2}$ = 0.44, but there was no significant interaction with group, *F*(12,264) = 1.37, *p* = 0.179, $\eta_{p}^{2}$ = 0.06. Altogether, participants scored highest on the dimension of attention to detail (Figure 8, right panel). Despite the null result at the group level, there was an indication of higher prevalence of autistic traits among some individual prodigies. The three highest AQ scores (i.e., 29, 33, and 34) belonged to prodigies and one late-trained musician and may indicate clinically significant levels of autistic traits (i.e., the cut-off AQ score is 32; Baron-Cohen et al., 2001). Indeed, the disorder was formally diagnosed in two participants with AQ scores of 29 and 33 (see section “Participants”).

**FIGURE 9:**
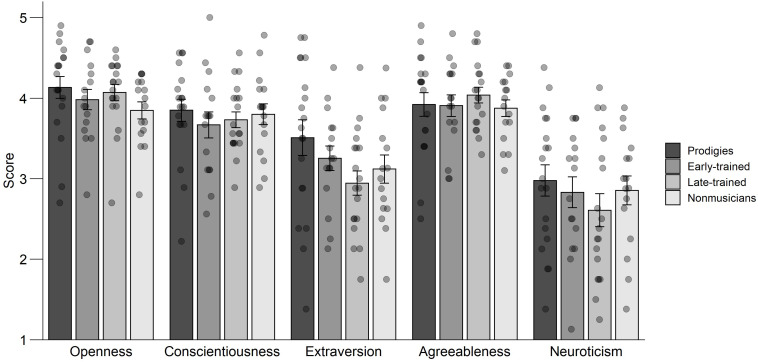
Big Five Inventory: mean scores, standard error and individual data, by trait and group. Points are jittered horizontally for visualization purposes.

For the Big Five Inventory, an ANOVA was computed on the mean score with group (prodigies, early-trained, late-trained, non-musicians) as a between-subjects factor and dimension or trait (openness to experience, conscientiousness, extraversion, agreeableness, and neuroticism) as a within-subject factor. The traits did not vary significantly by group, *F*(3,66) = 1.92, *p* = 0.135, $\eta_{p}^{2}$ = 0.08, and there was no interaction between group and traits, *F*(12,264) = 0.74, *p* = 0.715, $\eta_{p}^{2}$ = 0.03. However, there was a significant effect of trait, *F*(4,264) = 44.66, *p* < 0.001, $\eta_{p}^{2}$ = 0.40. Overall, participants tended to rate their openness, agreeableness, and conscientiousness high, and their extraversion and neuroticism low (Figure 9).

### Intellectual Quotient

**FIGURE 10:**
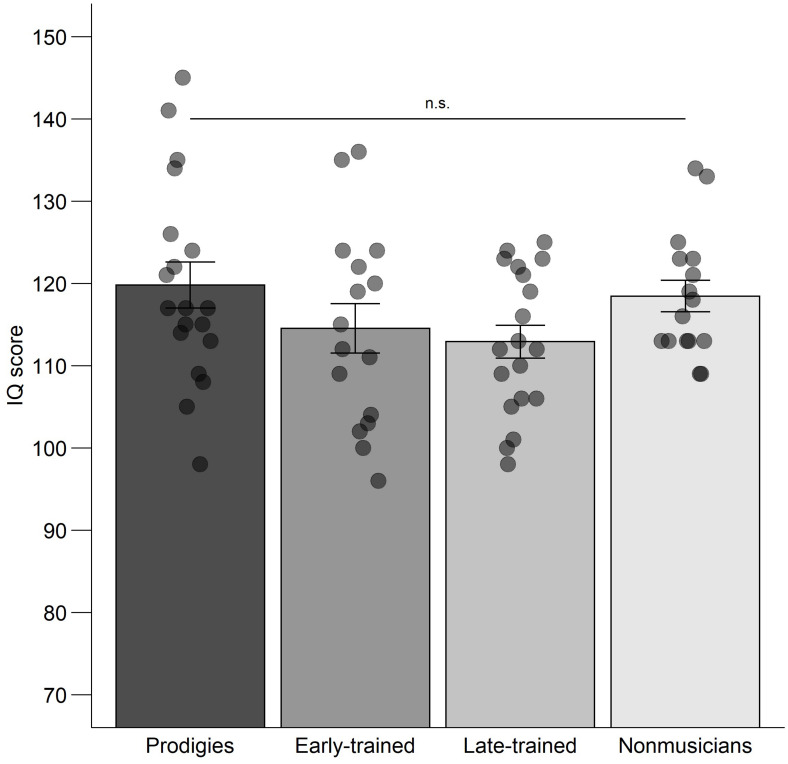
Global IQ: mean scores, standard error and individual data, by group. Note that the mean score in the general population is 100 and one standard deviation is 15 points. Points are jittered horizontally for visualization purposes.

Group mean IQ ranged from 113 to 120, which are above average but not exceptionally high considering that 95% of the adult participants had a university education. An ANOVA was computed on global IQ with group (prodigies, early-trained, late-trained, and non-musicians) as a between-subjects factor. There was no significant difference between groups, *F*(3,66) = 1.78, *p* = 0.159, η^2^ = 0.075 (Figure 10), nor between musicians (*M* = 116) and non-musicians (*M* = 118), *t*(36.21) = 1.08, *p* = 0.288.

**FIGURE 11:**
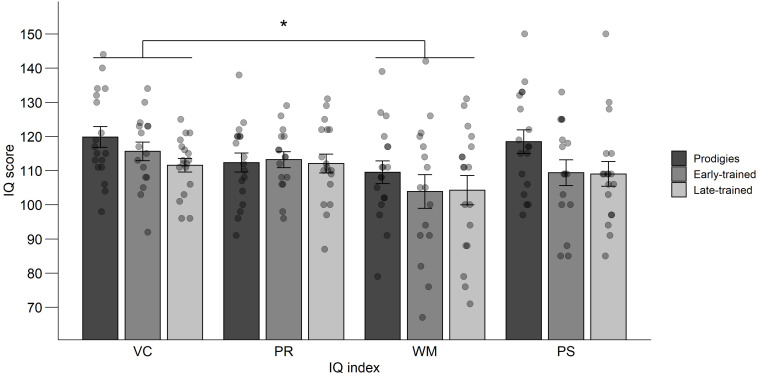
Mean IQ scores, standard error and individual data for verbal comprehension (VC), perceptual reasoning (PR), working memory (WM), and processing speed (PS), by group. Points are jittered horizontally for visualization purposes.

The IQ battery completed by musician participants included indices of verbal comprehension, perceptual reasoning, auditory-verbal working memory, and processing speed (Figure 11). An ANOVA was computed on the standardized individual index scores (*M* = 100, SD = 15, in the general population), with group (prodigies, early-trained, late-trained) as a between-subjects factor and index (verbal comprehension, perceptual reasoning, auditory-verbal working memory, and processing speed) as a within-subject factor. It revealed that verbal comprehension was better than working memory across groups, *F*(3,147) = 6.00, *p* < 0.001, $\eta_{p}^{2}$ = 0.11. The expected superiority of the prodigies was not significant in any index, as there was no group effect, *F*(2,49) = 1.99, *p* = 0.147, $\eta_{p}^{2}$ = 0.08, nor interaction between group and index, *F*(6,147) = 0.75, *p* = 0.607, $\eta_{p}^{2}$ = 0.03.

Visuo-spatial working memory, which was measured in all participants (grand mean = 19.5 of 26 trials, SD = 2.86), also did not differ according to group, *F*(3,65) = 1.11, *p* = 0.350, η^2^ = 0.049, as revealed by an ANOVA with group (prodigies, early-trained, late-trained, non-musicians) as a between-subjects factor.

### Correlation With Early Musical Practice

**FIGURE 12:**
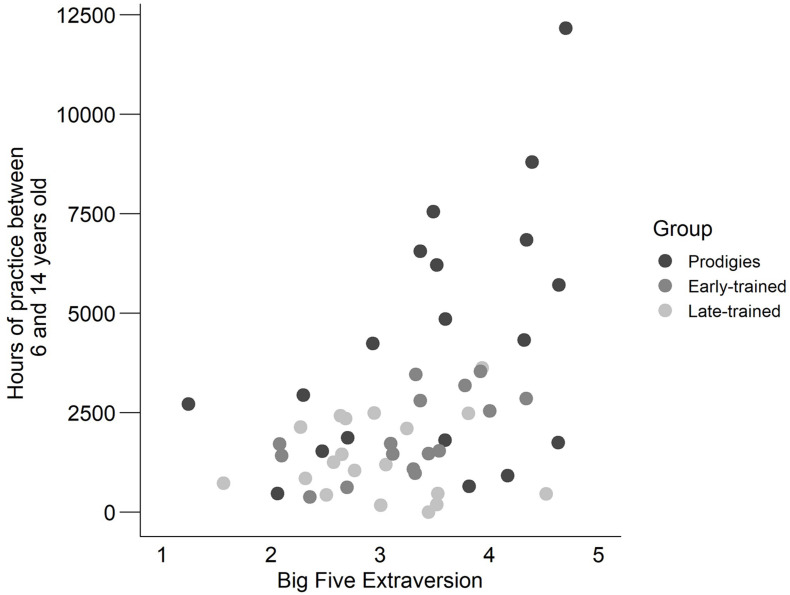
Deliberate practice accumulated between 6 and 14 years old in relation to extraversion as measured by the Big Five Inventory, by group.

Because early intensive practice is one of the factors that differentiated prodigies from the other musicians, we explored whether the individual amount of accumulated hours between age 6 and 14 was related to psychological traits measured here (i.e., 10 correlations; *p*-values adjusted using Bonferroni-Holm): global IQ, working memory index, processing speed index, openness to experience, conscientiousness, extraversion, music reward (BMRQ total score), autistic traits (AQ total score), attention to detail and flow. Individual amount of early practice varied considerably, especially among prodigies as mentioned previously, varying from 468–12,160 h accumulated between 6 and 14 years old. By comparison, early-trained musicians reported a range of 378–3,536 h and late-trained reported 0–3,623 h. The only trait to correlate significantly with the rate of early practice was extraversion, a dimension from the Big Five Inventory of personality, *r*(52) = 0.47, *p* = 0.004. While it appears at first glance that prodigies drive the correlation (Figure 12), separate correlation tests with only the prodigies (*r*(17) = 0.46, *p* = 0.048 [uncorrected]) or with only the non-prodigies (early-trained and late-trained musicians; *r*(33) = 0.35, *p* = 0.040 [uncorrected]), were significant as well. In other words, extraversion is generally correlated with amount of early practice.

## Discussion

The current research examined the lifetime accumulated practice and psychological traits of musical prodigies to identify markers of their exceptionality (as described in the Multifactorial Gene-Environment Interaction Model; MGIM). Prodigies were compared with non-prodigies who began their musical training similarly early (around age 6), or later (around age 10), and non-musicians. Unlike previous studies of prodigies (e.g., Ruthsatz et al., 2014; Ruthsatz and Urbach, 2012; Comeau et al., 2018), our large sample of prodigies did not differ from other musicians in terms of intelligence, working memory, or personality, including autistic traits. Around half of the prodigies reported having absolute pitch, but the proportion of reported absolute pitch possessors did not vary significantly across groups. The characteristics that set prodigies apart were their report of more frequent practice early in life, a more external motivation to begin playing their instrument, as well as a higher tendency to experience flow during practice. Thus, models such as the MGIM are more appropriate to describe the prodigy phenomenon than the deliberate practice view. Moreover, these results suggest that prodigies are at the high extreme of the continuum of musicality rather than constituting a distinct category of musicians. Prodigies did not differ from the controls on most variables, and when they did differ, as in tendency to experience flow when practicing, their scores overlapped greatly with the ones of the other musicians.

Prodigies reported practicing twice as much as their peers from the age of 6 to 14. However, contrary to what could be expected by the deliberate practice view (Ericsson et al., 1993), there was substantial variability. Some prodigies did not practice more than their peers (Figure 4) and nevertheless reached higher levels of achievement. Our data also indicate that prodigies practice as much as late-trained musicians when measured from the onset of their musical experience. This means that when they begin to play their instrument, prodigies practice as much as children who are around 5 years older than themselves. Different factors could explain this phenomenon. For example, since in general, older children can better sustain their attention (Lin et al., 1999), it could be an indication that prodigies have a more advanced development of sustained attention. We speculate as well that, when they start to play, late-trained musicians must ‘catch-up’ to the other musicians who have already started, especially if they want to pursue a musical career. Musicians who start to play later in life might not have shown early signs of musical aptitude (i.e., predispositions) or a particular interest toward music. Those predispositions to easily learn music could also explain the fact that some of the prodigies did not practice more than their age-matched peers and still managed to reach exceptional levels of achievement early on.

Obviously, amount of practice is no guarantee of quality, and in fact there was considerable variability of early practice even in prodigies (Figure 4). Musicians’ practice on a piece, for example, does not determine the evaluation of a newly learned piece by a jury (Williamon and Valentine, 2000). We propose that rate of progress would be a better index of the quality of practice. In our prior study of prodigies (Comeau et al., 2017), we noted that prodigies learned twice as fast as their peers, judging from the difficulty of musical pieces. For example, after 2.5 years of training, the prodigy Sarah Chang was capable of learning to play the Mendelssohn concerto on the piano whereas the typical pianist would only be capable after 10 years of training (Gagné and McPherson, 2016). Thus, prodigies not only practice more than their peers early on, but they also make more efficient use of their practice time.

Interestingly, prodigies reported that their source of motivation when beginning to play their instrument was more external compared to late-trained musicians, with early-trained musicians not significantly differing from either. Four prodigies but no early or late-trained controls reported the motivation being completely external (i.e., maximal rating). Parental investment might be one of the ingredients for fostering prodigiousness, but the relationship requires further study. For instance, parents may invest more time in response to the unusual behavior of their child. Highly invested parents have been suggested as playing a role in the development of their child’s exceptional abilities (Feldman, 1993), but prodigies are also characterized as having an exceptional inner drive to master their work (Winner, 2000). The use of more comprehensive measures of motivation, for example the complete questionnaire from which we selected individual questions (Desrochers et al., 2006) or interviews with children and parents, could help clarify the sources of motivation in young prodigies and musicians.

Besides parental influences, other factors may account for their distinctive practice behavior. Prodigies were more likely to report flow during musical practice compared to early-trained musicians. Since practice requires high levels of concentration, which is hard to maintain for young children (Lin et al., 1999), any factor that influences the inherent pleasure of the activity could influence motivation to continue. Future research could measure experience of flow directly after a practice session, as well as physiological correlates (Harmat et al., 2015), rather than self-report as used here (Butkovic et al., 2015).

Autistic traits are associated with genetic factors (Miles, 2011), yet autism does not seem to characterize most musical prodigies. Only two of the 19 prodigies met the criteria for clinically significant autistic traits based on their responses to the Autism Spectrum questionnaire (Baron-Cohen et al., 2001) or formal diagnosis. Thus, we found no evidence that autism is a relevant candidate disorder in the search for common genes explaining exceptional achievements. Personality, in contrast, may play a small role. We found that the more a child practiced before adolescence, the more extraverted they reported to be. Extraversion might influence practicing indirectly because it could motivate participation in stage arts (Ullén et al., 2016). However, we note that success in competitions was a selection criterion used here and elsewhere (Ruthsatz and Urbach, 2012) for considering a child as a musical prodigy and musical prodigies were no more extraverted than other participants in our sample.

The early advantage in learning for prodigies appears to be limited to music. We found no evidence of superior intelligence or exceptional working memory in prodigies compared to other musicians, nor did we observe heightened cognitive abilities in musicians compared to non-musicians. The latter finding is in line with a recent meta-analysis obtaining no evidence for a causal effect of musical training on general cognitive abilities (Sala and Gobet, 2020). Even though most prodigies in our sample (68%) were tested as adults, age of testing does not necessarily undermine the findings. Longitudinal studies show stability of IQ scores from 6 years onward (see Yu et al., 2018; for a review).

In summary, we found that early intense practice characterizes musical prodigies during early childhood, a time when the brain is most plastic (Herholz and Zatorre, 2012). Because pre-existing differences in the recruitment of brain regions involved in auditory encoding and motor control predict success in learning to play an instrument, we may expect prodigies to be born with pre-existing differences in these brain networks. Such predispositions may be amplified by early and sustained practice. Future research should aim to identify the anatomical and functional properties of brain networks that affect exceptional learning rate and achievement. Researchers should also try to recruit prodigies while they are children, in order to better measure the traits and behaviors associated with the prodigy phenomenon as it unfolds.

## Data Availability Statement

The raw data supporting the conclusions of this article will be made available by the authors, upon reasonable request.

## Ethics Statement

The studies were reviewed and approved by the Comité d’Éthique de la Recherche en Arts et en Sciences (CÉRAS), University of Montreal, Montreal, Canada. Written informed consent to participate in this study was provided by the participants or their legal guardian/next of kin.

## Author Contributions

CM and IP contributed equally in the project’s conception. CM, MS, and IP participated in the study design. CM performed the literature search and drafted the manuscript. CM and MW performed the statistical analysis. MW and MS provided the critical revisions. IP performed the final revisions. All authors contributed to the article and approved the submitted version.

## Conflict of Interest

The authors declare that the research was conducted in the absence of any commercial or financial relationships that could be construed as a potential conflict of interest.
