# Supplementary material for: What Makes Musical Prodigies?
Source: Front Psychol. 2020 Dec 11;11:566373. doi: 10.3389/fpsyg.2020.566373 (PMC7759486; doi:10.3389/fpsyg.2020.566373)
Supplement: Supplementary file 1 [file Table_1.docx]

Supplementary Material

**Supplementary table 1.** Definitions of prodigies with Author in parentheses.

| “a child (typically younger than 10 years old) who is performing at the level of a highly trained adult in a very demanding field of endeavor.” | (Feldman, 1993) |
| --- | --- |
| “Prodigies are able to function at an advanced adult level in some domain before age 12.” | (Solomon, 2012) |
| “a child who, at onset of puberty (which for boys is usually 11–12 and girls 10–11), manifests extraordinary intellectual–creative performance and/or achievements in any type of a real activity (i.e. intellectual, artistic, musical, and so on).” | (Shavinina, 2016) |
| “Gifted children and prodigies display near-adult level skills and interests.” | (Winner, 2000) |
| “The current study deemed a child to have prodigious talent  if he or she had achieved national or international acclaim by adolescence.” | (Ruthsatz, Ruthsatz, & Stephens, 2014) |
| “Prodigies are children under 10 years of age who perform culturally relevant tasks at a level that is rare even among highly trained professionals.” | (Ruthsatz & Detterman, 2003) |
| “Prodigies are children who display exceptional talent early in life” | (McPherson & Hallam, 2009) |

**Supplementary table 2.** Prodigies’ age for each type of achievement.

| **Subject** | **First prize in national or international competition** | **Second prize in national or international competition or first prize in regional competition** | **Special recognition of their talent** |
| --- | --- | --- | --- |
| **P1** |  | 14, 15, 16, 16 | 12: Orchestral debut 18: Admission to Juilliard |
| **P2** |  | 8, 9, 10 |  |
| **P3** | 12, 15 | 9, 10 |  |
| **P4** | 9, 12, 13, 17 |  | 8: Television appearance |
| **P5** |  | 18 | 10: Television appearance |
| **P6** |  | 10, 13, 14, 15, 16, 17 |  |
| **P7** |  |  | 9: Television appearance |
| **P8** | 13 | 15, 16 | 14: Television appearance 16: Television appearance |
| **P9** |  | 13 | 12: Youngest concertmaster of the school’s orchestra 13: Television appearance |
| **P10** | 14 |  | 12: Documentary appearance 16: Chosen to be in the YouTube symphony orchestra 17: Newspaper article 18: “Revelation of the year” by national television |
| **P11** | 12, 16, 17 | 12, 12 |  |
| **P12** | 9, 14 | 10, 11, 12, 14 |  |
| **P13** |  | 11, 12, 14 |  |
| **P14** | 16, 16 | 18 | 7: Orchestral debut 12: Invited to perform with professional orchestra |
| **P15** |  | 10, 11, 12, 12 | 10: 1^st^ violin of youth orchestra 13: Winner of national television competition |
| **P16** |  | 8, 9 | 11: Television appearance |
| **P17** | 9, 10, 11, 13, 16, 17 |  |  |
| **P18** | 9 |  |  |
| **P19** | 10, 10, 12 |  | 13: Public’s favorite prodigy at an international competition |


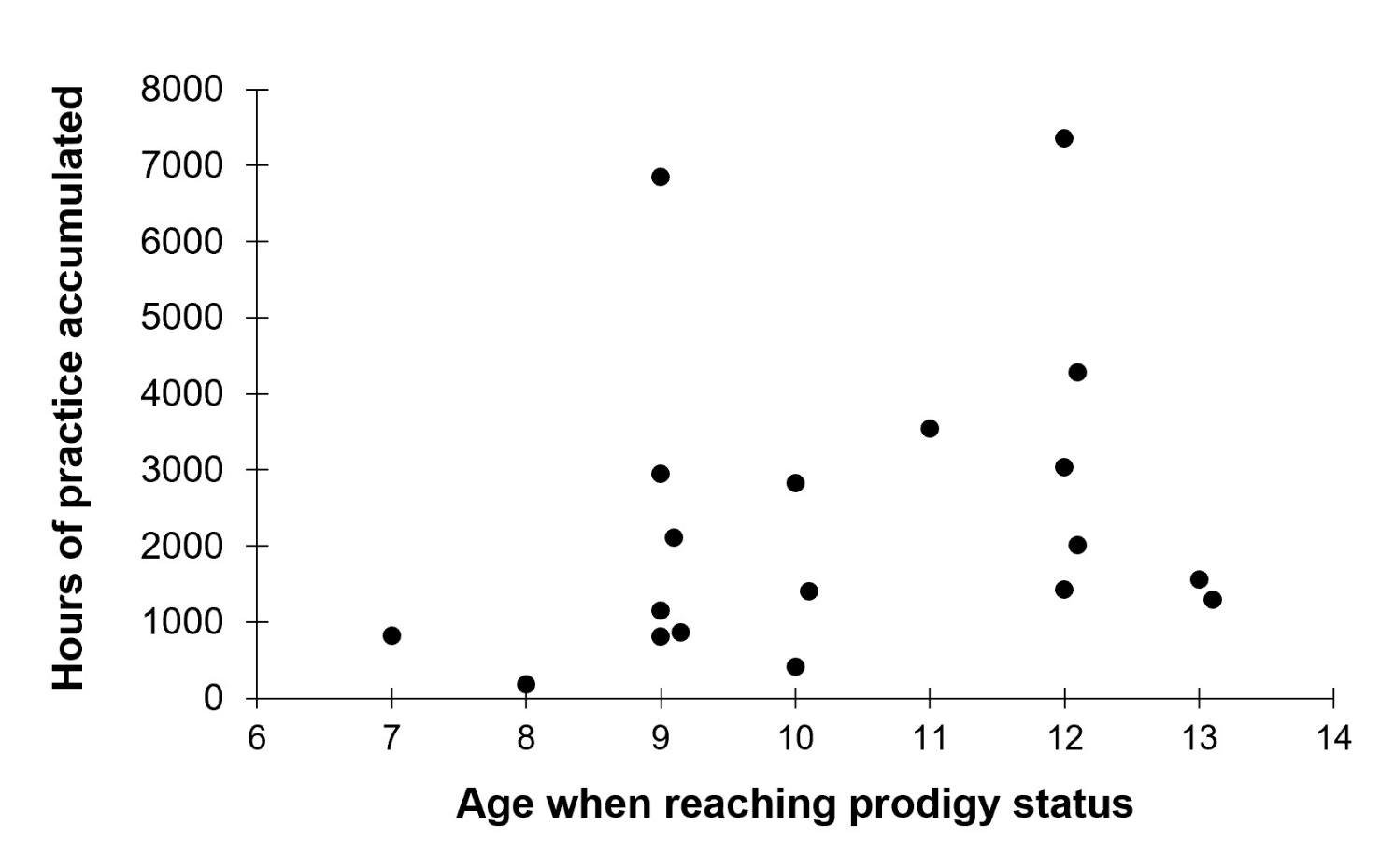


**Supplementary figure 1.** Age when reaching prodigy status and hours of practice accumulated by that age.
